# Loneliness and Perceived Social Support in Endometriosis: The Roles of Body Image Disturbance and Anticipated Stigma

**DOI:** 10.1007/s12529-023-10230-w

**Published:** 2023-10-26

**Authors:** Catherine Calvi, Kerry A. Sherman, Dione Pham

**Affiliations:** 1 Lifespan Health and Wellbeing Research Centre, Macquarie University, 16 University Avenue, Sydney, NSW 2109 Australia; 2 School of Psychological Sciences, Macquarie University, Sydney, Australia; 3 Smart Green Cities Research Centre, Macquarie University, Sydney, Australia

**Keywords:** Chronic illness, Endometriosis, Body image, Stigma, Loneliness

## Abstract

**Background:**

Research has identified that living with the chronic inflammatory disease endometriosis adversely impacts social functioning and interpersonal relationships, specifically, feelings of loneliness and a lack of perceived social support. Commonly experienced body image disturbance (BID), combined with the anticipation of endometriosis-related stigma from others, may result in further social withdrawal. This study aimed to quantitatively investigate the association between BID and social functioning (loneliness and diminished perceived social support), and the potential moderating effect of anticipated stigma on these associations.

**Method:**

Participants (*N* = 212) with a self-reported endometriosis diagnosis completed an online questionnaire measuring social and emotional loneliness, perceived social support, BID, anticipated stigma and demographic and medical characteristics.

**Results:**

Mean scores indicated high levels of BID, emotional loneliness and diminished perceived social support. Bootstrapped multivariable regression analyses indicated that BID was significantly associated with greater emotional loneliness and lower perceived social support. BID was also associated bivariately with greater social loneliness. Anticipated stigma from healthcare workers moderated the association of BID with perceived social support, such that poorer perceived support was reported when anticipated stigma was high, despite the presence of minimal BID.

**Conclusion:**

These findings highlight the psychological challenges of living with endometriosis in terms of highly prevalent BID, in the context of feeling lonely and poorly supported. The further negative impact of anticipated stigma suggests that psychosocial interventions may benefit from additionally targeting these perceptions of stigma.

**Supplementary Information:**

The online version contains supplementary material available at 10.1007/s12529-023-10230-w.

## Introduction

Estimated to occur in one in nine people assigned female at birth [1], endometriosis is a chronic inflammatory condition characterised by chronic pelvic pain, fatigue, dysmenorrhoea (painful menstruation), dyspareunia (pain associated with sexual intercourse), nausea, and bowel and bladder issues [2]. Individuals living with endometriosis (ILWE) endure extensive diagnostic delays of approximately 6 to 8 years following symptom onset [3, 4]. There is no known cure, and severe symptoms commonly reoccur even after undergoing invasive surgical procedures [5].

Emerging research in endometriosis suggests that the experience of living with this chronic condition may lead to difficulties with social functioning, particularly feelings of loneliness and being poorly supported by others [6–8]. Qualitative investigations have documented significant physical barriers to social activities for ILWE, including chronic pain and fatigue, as well as the anticipated need to urgently access bathroom facilities due to nausea, bowel and bladder issues and/or heavy menstrual bleeding [9, 10]. Subsequently, many ILWE choose to restrict the size of their social networks to be more manageable [11], particularly younger individuals which consequently may lead to feeling alienated from their peers [10]. Endometriosis also impacts romantic relationships, with half of participants in a large cross-cultural sample reporting their relationships had been adversely affected by their condition [12]. Being isolated from peers and social activities due to endometriosis symptoms is likely to lead to feelings of loneliness, a subjective emotional experience occurring when there is a perceived deficiency in the quality or quantity of one’s relationships, as compared to a subjective ideal [13]. By this definition, loneliness goes beyond social isolation, since it is possible to have limited social contact and not be lonely [14]. Due to the high symptom burden and constraints endometriosis places on social activities and everyday functioning [6, 10], ILWE are likely to experience both social (i.e. a perceived deficit in either the size of, or embeddedness in, one’s social network) and emotional (i.e. the absence of an attachment figure or a conflict-ridden romantic relationship) loneliness [13, 15]. As such, this project aims to investigate both aspects of loneliness in ILWE.

Another social functioning-related concern for ILWE identified in quantitative [10, 16] and qualitative [9] investigations is a perceived lack of social support. This coping resource can serve as a buffer against distress, and when social support is absent, this may result in loneliness and diminished wellbeing [17]. The many years ILWE often spend living with symptoms of endometriosis in the absence of a formal diagnosis further erodes perceptions of being supported [6]. Conversely, a diagnosis provides a sense of legitimization and language through which ILWE can communicate with others and subsequently access social supports [18].

Body image, defined as thoughts, feelings, beliefs and behaviours regarding one’s body appearance and functionality [19], is another factor likely impacting social functioning in ILWE. Chronic pain, functional impacts and/or aesthetic changes associated with endometriosis fuel the commonly experienced adverse impact of body image disturbance (BID) [20, 21], characterised by a persistent preoccupation and distress associated with one’s body image [22]. According to White’s [23] cognitive behavioural model, these illness-induced bodily changes activate negative automatic thoughts, appearance-related assumptions, maladaptive behaviours and emotional distress, which, in turn, maintain negative self-beliefs about one’s body. In qualitative research, ILWE have expressed frustration and feelings of hopelessness and loss of confidence over the limitations and changes that endometriosis imposes on their bodies [24, 25]. Bloating and weight gain, particularly during symptom ‘flare ups’, leave ILWE unable to wear their preferred clothes, both to avoid physical discomfort and to conceal bodily features considered embarrassing and socially undesirable [5, 25].

Prior research in endometriosis [26] and non-endometriosis [27–29] populations suggests there is likely a strong association between BID and impaired social functioning, including feelings of loneliness and a perceived lack of social support. This link between BID and social functioning may be explained in part by objectification theory, whereby individuals are said to be socialised to internalise appearance-related feedback from others that occurs in interpersonal encounters [30]. This internalisation and resulting expectation of negative appearance-based feedback can induce self-consciousness in social interactions that depletes cognitive resources, impedes engagement and motivates social withdrawal to avoid further perceived criticism [30]. Qualitative evidence regarding ILWE is consistent with this theory in that appearance-related shame and embarrassment are suggested to drive concealment practices and hypervigilance in social situations [5], and the tendency to socially withdraw as a coping mechanism [11]. Furthermore, mediational analyses have demonstrated that BID in ILWE leads to diminished self-esteem [21], which may adversely impact the initiation and maintenance of social relationships [31] and therefore compromise social functioning.

The relationship between BID and diminished social functioning in ILWE may be exacerbated by anticipated stigma, which, in a chronic illness context, is defined as fears of negative evaluations or rejection by others based on their condition [5, 32]. For ILWE, stigma associated with endometriosis as a condition is further compounded by the ‘social etiquette’ that accompanies menstruation, such that it is something to be endured privately [33]. Anticipated stigma acts as a barrier for seeking medical care [34], with ILWE experiencing widespread trivialisation of severe symptoms by healthcare providers, leaving them feeling isolated and doubting their own accounts of pain [33, 35]. Moreover, qualitative evidence documents a reluctance to disclose endometriosis-related distress to others to avoid being labelled weak or emotional [5, 11]. This concealment of distress is evident in the practice of self-silencing, which refers to a lack of assertion of needs in close relationships to avoid making others uncomfortable or out of fear of losing relationships [36]. Subsequently, the individual may feel unsupported and isolated as their needs are not being heard [11]. Hence, as ILWE experience BID, they may additionally feel more lonely and less socially supported if they also feel as if they cannot communicate this distress to close others out of fears of disrupting these relationships. Although the pervasive impacts of stigma have been well documented in other chronic illness populations [37, 38], quantitative data are lacking for ILWE [39].

## The Present Study

**Fig. 1:**
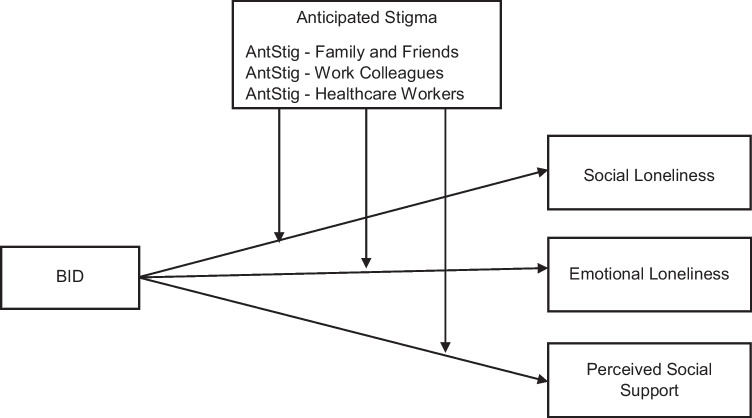
Graphical representation of hypothesised relationships between the variables of interest

This study aimed to extend prior qualitative evidence by quantitatively investigating the association between BID and social functioning in terms of feelings of loneliness and diminished perceived social support (see Fig. 1). The potential moderating effect of anticipated stigma on these associations was also investigated. Each of these key constructs has been individually identified as a significant psychosocial issue among ILWE [16, 39]; however, this is the first known study to examine the associations between these variables in this population. It was hypothesised that greater BID would be associated with increased feelings of (H1) social and (H2) emotional loneliness, and that anticipated stigma would moderate (strengthen) these relationships (H3a and H3b, respectively). It was also hypothesised that greater BID would be associated with lower perceived social support (H4), and that this relationship would be moderated (strengthened) by anticipated stigma (H5).

## Method

### Participants and Procedure

This cross-sectional survey design study was conducted in June to July of 2022 as part of a larger research project investigating social functioning in ILWE. Ethical approval for this project was obtained from the Macquarie University Human Research Ethics Committee (Reference: 520221140337599), and the study protocol was preregistered on the Open Science Framework (https://doi.org/10.17605/OSF.IO/B8YNV). All procedures performed in this study were in accordance with the ethical standards of the institutional research committee and with the 1964 Helsinki Declaration and its later amendments. The sample was recruited from the social media networks of the Australian organisation Endometriosis Australia. Inclusion criteria were as follows: being at least 18 years old, self-reporting a previous clinical or surgical diagnosis of endometriosis, having internet access and having English language competency to complete the online survey. No compensation was provided for study participation. Data were collected and managed using the secure, electronic platform REDCap (Research Electronic Data Capture) [40, 41] hosted at Macquarie University. This study is reported according to STROBE guidelines for cross-sectional data.

**Fig. 2:**
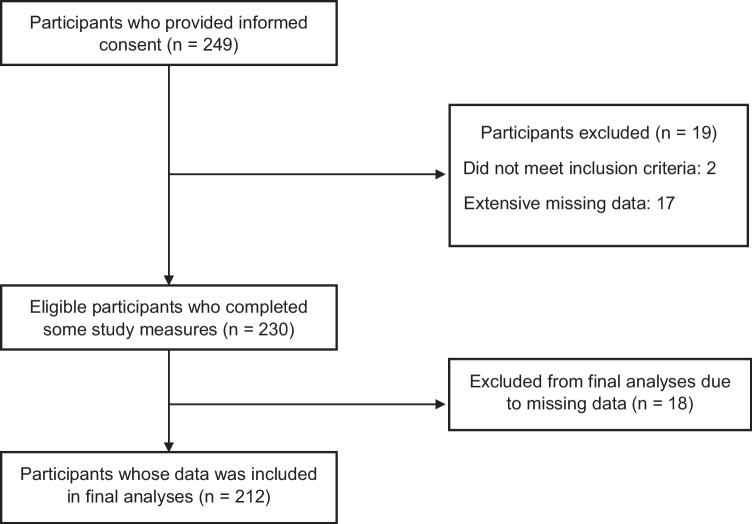
Flow of participants through the study

Participants who self-reported meeting eligibility criteria accessed the online consent form and survey via the REDCap weblink. Following the provision of informed consent, participants completed survey questions assessing demographics, medical information and psychological and interpersonal measures. In total, 249 individuals provided online informed consent, of which 37 were excluded due to either extensive missing data or not meeting inclusion criteria. The final sample contained 212 participants who provided sufficient data to be included in final analyses (see Fig. 2). Participant characteristics are displayed in Table 1. A power analysis conducted using G*Power ver. 3.1 [42] indicated a sample size of at least 133 for a small to medium effect size to achieve a power of 0.95 at the 0.05 significance level for linear multiple regression analyses.

**Table 1:** Sample demographic characteristics and bivariate associations with dependent variables

Demographic characteristic	Total sample (*N* = 212^a^)	Association with dependent variables		
		Lon_Soc	Lon_Emot	Soc_Support
		*r*_s_ or *H*	*r*_s_ or *H*	*r*_s_ or *H*
Age (years)^b^, *M* (SD)	31.61 (7.50)	0.09	0.01	− 0.10
Aboriginal or Torres Strait Islander^c^, *n* (%)	4 (1.90)	0.003	0.21	0.05
Gender identity^c^, *n* (%)				
Woman	206 (98.58)	2.11	1.51	4.82
Transgender woman	1 (0.47)			
Non-binary	1 (0.47)			
Gender fluid	1 (0.47)			
Has children^c^, *n* (%)	54 (25.47)	0.06	0.82	0.35
In a romantic relationship^c^, *n* (%)	161 (76.30)	1.28	3.14	1.14
Sexual orientation^c^, *n* (%)				
Heterosexual	175 (82.94)	1.55	7.07	1.94
Bisexual	17 (8.06)			
Queer	9 (4.27)			
Pansexual	3 (1.43)			
Lesbian	2 (0.95)			
Asexual	1 (0.48)			
Prefer not to say	4 (1.90)			
Country of birth^c^, *n* (%)				
Australia/NZ	178 (86.41)	2.10	1.31	3.51
USA	3 (1.46)			
UK/Europe	11 (5.34)			
South America	7 (3.40)			
Asia	7 (3.40)			
Living in Australia^c^, *n* (%)	201 (95.26)	0.01	0.12	0.00
Living outside a metro. area^c^, *n* (%)	82 (38.68)	0.49	2.72	4.66*
Education completed^b^, *n* (%)				
High school or less	36 (17.06)	− 0.03	− 0.15*	− 0.19**
Vocational/other tertiary education	47 (22.27)			
Undergraduate degree	70 (33.18)			
Postgraduate degree	58 (27.49)			
Employment^c^, *n* (%)				
Full time	109 (51.42)	2.79	2.78	7.64*
Part time/casual	59 (27.83)			
Not working	21 (9.91)			
Student/home duties	23 (10.85)			
Diagnosis method^c^, *n* (%)				
Surgery	191 (91.47)	1.88	0.04	1.81
Ultrasound/MRI	14 (6.64)			
Based on clinical symptoms	4 (1.90)			
Diagnostic delay (years)^b^, *M* (SD)	8.89 (5.97)	0.01	0.01	0.06
Perceived endometriosis severity^b^, *M* (SD)	2.32 (0.65)	0.06	0.13	0.20**
Total no. of symptoms^b, e^, *M* (SD)	6.99 (1.78)	0.18**	0.20**	0.17*
Pelvic pain, *n* (%)	202 (95.28)			
Abdominal bloating, *n* (%)	176 (83.02)			
Abdominal cramping, *n* (%)	188 (88.68)			
Fatigue, *n* (%)	201 (94.81)			
Nausea/vomiting, *n* (%)	122 (57.55)			
Treatment^c, d, e^, *n* (%)				
Surgery	130 (61.32)	0.02	0.01	3.30
Hormonal medication	127 (59.91)	2.74	2.78	0.39
Pain medication	164 (77.36)	1.20	1.32	9.85**
Complementary therapies	11 (5.19)	0.78	0.04	0.24
Physiotherapy	6 (2.83)	0.56	0.45	0.03
PANAS-NA^b^, *M* (SD)	22.52 (8.71)	0.13	0.26**	0.39**

*r*_s_ is Spearman’s rank correlation coefficient, *H* is Kruskal-Wallis *H* statistic, Lon_Soc is the social loneliness subscale of the De Jong Gierveld Loneliness Scale (DJGLS), Lon_Emo is the emotional loneliness subscale of the DJGLS, Soc_Support is the social support subscale of the Endometriosis Health Profile 30 and PANAS-NA is the negative affect subscale of the Positive and Negative Affect Schedule

*MRI* magnetic resonance imaging

*p < .05; **p < .01

^a^Sample size varies due to incomplete data

^b^Spearman’s rank correlation coefficient was used due to non-normal distribution

^c^Kruskal-Wallis *H* test was used due to non-normal distribution

^d^Categories not mutually exclusive

^e^Referring to the previous 12 months

### Measures

The 11-item De Jong-Gierveld Loneliness Scale (DJGLS) [43] assessed social loneliness (e.g. “there are enough people I feel close to”) and emotional loneliness (e.g. “I often feel rejected”, “I miss having a really close friend”). Total scores range from 0 to 5 (social) and from 0 to 6 (emotional), with higher scores indicating greater feelings of loneliness. The DJGLS demonstrates convergent validity [44] and stability across several cultural contexts [45]. Internal consistency for the current sample (*α*_social_ = .79, *α*_emotional_ = .85) was satisfactory.

The Social Support Subscale of the Endometriosis Health Profile 30 (EHP-30) [46] assessed perceived social support (e.g. “Felt as though others think you are moaning?”, “Felt unable to tell people how you feel?”). Developed using interviews with ILWE, the EHP-30 is a frequently used endometriosis-specific quality of life measure, demonstrating satisfactory item reliability and convergent validity [47]. Summed scores of the 4-item subscale are converted to a total score out of 100, representing the lowest level of perceived social support. The social support subscale displayed good internal consistency in the present study (*α* = .87).

The Body Image Scale (BIS) [48] assessed the cognitive, behavioural and affective aspects of body image (e.g. “Have you been satisfied with your appearance when dressed?”, “Have you been feeling self-conscious about your appearance?”). Originally developed in an oncology context, it has since been utilised in endometriosis [21]. One item was removed regarding feelings of femininity to ensure gender inclusivity. The total summed score was then scaled to a possible total of 30 to be comparable to the original scale. Higher scores indicate poorer body image, and scores 10 and above denote likely clinically significant BID [49, 50]. The BIS has documented good test-retest reliability and discriminant validity in a benign gynaecological condition context [51]. Internal consistency in the current sample was high (*α* = .91).

Developed for individuals living with chronic illness, the Chronic Illness-Related Anticipated Stigma Scale (CIASS) [52] evaluated anticipated stigma from friends and family (FF), work colleagues (WC) and healthcare workers (HW). Items for each subscale include the following: “a friend or family member will think that your illness is your fault” (FF), “someone at work will think that you cannot fulfil your work responsibilities” (WC) and “a healthcare worker will be frustrated with you” (HW). Total mean scores for each 4-item subscale were calculated, with higher scores indicating greater anticipated stigma from the relevant source. The CIASS exhibits convergent validity and item reliability [52]. Internal consistency for each subscale was excellent (*α*_FF_ = .90, *α*_WC_ = .93, *α*_HW_ = .94) in the current sample.

#### Potential Covariates

Demographic details collected included age, Aboriginal or Torres Strait Islander status, gender identity, sexual orientation, whether they were in a romantic relationship, education completed, country of birth and residence, residing in a metropolitan location, employment status and parental status. Participants also self-reported endometriosis-specific medical information, including method of diagnosis, diagnostic delay (years between symptom onset and diagnosis) and perceived endometriosis severity (4-point Likert-type scale ranging from 0 [asymptomatic] to 3 [severe]), and self-reported symptoms currently experienced and treatments received in the last 12 months.

The 10-item Negative Affect subscale of the Positive and Negative Affect Schedule (PANAS) [53]  assessed the current emotional state of the participants. This was included as a covariate since negative emotional state may inflate the salience of negative memories and hence bias survey responses [54]. The PANAS exhibits good structural validity [55]. Internal consistency in the current sample was excellent (*α* = .90). 

### Statistical Analysis Plan

Descriptive statistics were used to examine the frequencies of categorical variables, and means and standard deviations of continuous data. Univariate distributions and Shapiro–Wilk statistics were inspected to determine normality, and non-parametric tests were utilised for analyses of non-normally distributed variables. Associations between the dependent variables and potential covariates were investigated to determine inclusion of covariates in further analyses. As all variables of interest were non-normally distributed, Spearman’s rho tests (continuous variables) and Kruskal-Wallis *H* tests (categorical variables) were conducted. To test study hypotheses, a series of bootstrapped multivariable linear regression analyses were undertaken, controlling for identified covariates.

## Results

Bivariate analyses identified several variables associated with each specific dependent variable that were subsequently included as covariates (see Table 1). Significantly positive correlations were found between all variables of interest (see Table 2). Specifically, regarding hypotheses 1 and 2, at the bivariate level BID was significantly correlated with both social loneliness (*r* = .28) and emotional loneliness (*r* = .41), respectively. In addition, regarding hypothesis 4, BID was significantly correlated with perceived social support (*r* = .57). Mean BID scores in the sample exceeded the suggested clinical cut-off of 10, with only 20% of scores falling below this value [50].

**Table 2:** Bivariate associations between variables of interest

Variables	1	2	3	4	5	6	7
1. Lon_Soc	–						
2. Lon_Emot	0.59**	–					
3. Soc_Support	0.30**	0.45**	–				
4. BIS	0.28**	0.41**	0.57**	–			
5. Stigma_FF	0.33**	0.37**	0.35**	0.37**	–		
6. Stigma_Work	0.25**	0.31**	0.41**	0.38**	0.49**	–	
7. Stigma_Health	0.14*	0.16*	0.44**	0.36**	0.48**	0.50**	–
*M*	1.50	3.45	70.42	16.52	2.41	3.05	2.98
SD	1.67	2.23	23.23	7.81	1.05	1.15	1.26
*n*	208	206	210	210	206	205	205

Stigma_FF is the family and friends subscale of the Chronic Illness Anticipated Stigma Scale (CIASS), Stigma_Work is the work colleagues subscale of the CIASS and Stigma_Health is the healthcare workers subscale of the CIASS

*BIS* Body Image Scale

*p < .05; **p < .01

All multivariable linear regression analyses are presented in Table 3. Regarding social loneliness (H1), the overall model was significant (*χ*^2^ (5, 203) = 24.30, *p* < .001, *R*_adj_^2^ = .09); however, there was no main effect for BID. There was a significant main effect for anticipated stigma from family and friends only (*R*^2^ = .04). Since the interaction terms between BID and anticipated stigma were non-significant, they were therefore removed from the final model (H3a; see Supplementary File). Regarding hypothesis 2, the overall model for emotional loneliness was also significant (*χ*^2^ (9, 199) = 84.05, *p* < .001, *R*_adj_^2^ = .21). Significant main effects were evident for BID (*R*^2^ = .06) and anticipated stigma from family and friends (*R*^2^ = .05). The three interaction terms between BID and anticipated stigma were non-significant (H3b) and were removed from the final model (see Supplementary File).

**Table 3:** Bootstrapped multivariable regression analyses

Variables	*b* (SE *b*)	BCA 95% CI for *b*		*z*	*χ*^2^	*R*_adj_^2^
		LL	UL			
Model 1: Lon_Soc					23.97**	.09
Total no. of symptoms	0.06 (0.07)	− 0.06	0.21	0.94		
BIS	0.03 (.02)	0.001	0.06	1.97		
Stigma_FF	0.36 (0.13)	0.10	0.61	2.76**		
Stigma_Work	− 0.13 (0.12)	− 0.10	0.36	1.09		
Stigma_Health	− 0.12 (0.10)	− 0.33	0.08	− 1.19		
Model 2: Lon_Emot					87.52**	.21
Education						
Vocational/Other tertiary education	− 0.06 (0.46)	− 0.99	0.84	− 0.13		
Undergraduate	− 0.48 (0.45)	− 1.35	0.43	− 1.06		
Postgraduate	− 0.10 (0.49)	− 1.05	0.87	− 0.20		
Total no. of symptoms	0.04 (0.09)	− 0.12	0.22	0.46		
PANAS_Na	0.02 (0.02)	− 0.01	0.05	1.09		
BIS	0.08 (0.02)	0.04	0.12	3.75**		
Stigma_FF	0.50 (0.16)	0.19	0.81	3.17**		
Stigma_Work	0.25 (0.16)	− 0.08	0.56	1.57		
Stigma_Health	− 0.21 (0.14)	− 0.49	0.06	− 1.52		
Model 3: Soc_Support					153.04**	.42
Outside metro. area	3.57 (2.70)	− 1.56	8.81	1.34		
Education						
Vocational/Other tertiary education	1.32 (4.35)	− 7.28	9.76	0.30		
Undergraduate	− 1.72 (3.77)	− 8.91	5.84	− 0.46		
Postgraduate	− 0.34 (3.65)	− 7.44	7.00	− 0.09		
Employment						
Part time/casual	− 1.22 (2.89)	− 5.68	5.54	− 0.04		
Not working	− 2.17 (4.09)	− 10.81	5.20	− 0.53		
Student/home duties	− 4.46 (4.38)	− 12.86	4.16	− 1.02		
No. of symptoms^a^	0.14 (0.83)	− 1.44	1.76	0.17		
Endometriosis severity^a^	0.39 (2.49)	− 4.47	5.28	0.15		
Pain medication^a^	4.80 (3.74)	− 2.39	12.15	1.28		
PANAS_Na^a^	0.33 (0.15)	0.02	0.63	2.12*		
BIS^a^	1.02 (0.22)	0.59	1.44	4.64**		
Stigma_FF^a^	0.46 (1.55)	− 2.53	3.52	0.30		
Stigma_Work^a^	2.26 (1.43)	− 0.47	5.09	1.58		
Stigma_Health^a^	4.20 (1.31)	1.66	6.79	3.22**		
BIS × Stigma_Health^b^	− 0.30 (0.14)	− 0.56	− 0.02	− 2.18*		

SE *b* is the bootstrapped standard error for *b*, BCA 95% CI is the bias-corrected and accelerated 95% confidence interval, Outside metro. area means living in regional/rural/remote area and BIS × Stigma_Health is the interaction term between BIS and Stigma_Health

*LL* lower limit, *UL* upper limit

*p < .05; **p < .01

^a^Numeric variables were mean centred to aid the interpretation of main effects

^b^Interaction term constructed using mean-centred variables

**Fig. 3:**
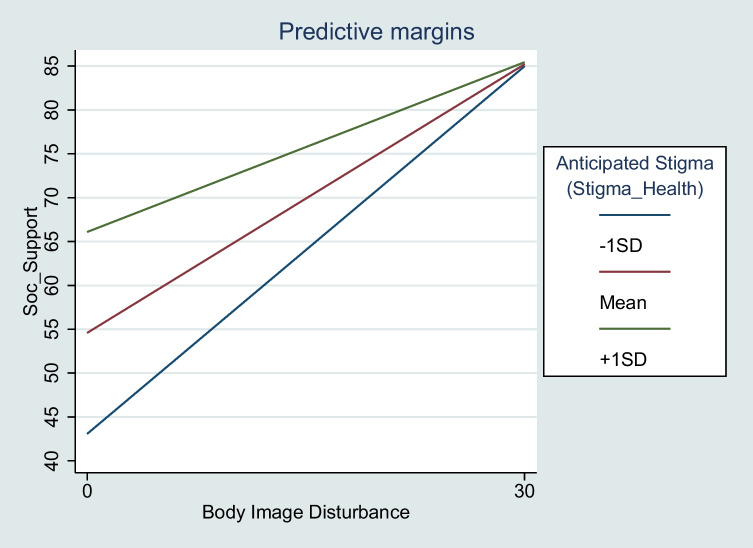
Simple slopes analysis of the interaction between body image disturbance and anticipated stigma from healthcare workers on perceived social support

Regarding hypothesis 4, perceived social support, the overall model was significant (*χ*^2^ (16, 202) = 156.02, *p* < .001, *R*_adj_^2^ = .42). Significant main effects were evident for BID (*R*^2^ = .1) and anticipated stigma from healthcare workers (*R*^2^ = .06). The BID × anticipated stigma from healthcare workers interaction was significant (H5), accounting for 2.49% of unique variance in perceived social support (see Fig. 3) such that for low BID and low anticipated stigma (− 1 SD), perceptions of social support are high (*B* = 1.39, *z*(202) = 4.88, *p* =  <.001). However, the protective effect of low BID on perceived support is negated for those with greater anticipated stigma (+ 1 SD), who perceive poorer social support (*B* = .65, *z*(202) = 2.36, *p* = .02). Conversely, when BID is high, perceptions of social support are poor irrespective of anticipated stigma. The remaining interaction terms were non-significant (H5; see Supplementary File).

## Discussion

This study aimed to investigate the relationships between BID and impairments in social functioning in endometriosis, specifically feelings of loneliness and perceived social support, and to examine the potential moderating role of anticipated stigma on these relationships. As predicted, BID was associated with greater emotional loneliness and less perceived social support, although the association between BID and greater social loneliness was only significant at the bivariate level. Moderation analyses revealed that for those with low BID scores, perceived social support was significantly poorer for those with greater anticipated stigma from healthcare workers, whereas high BID was associated with poor perceived social support, regardless of anticipated stigma.

On average, study participants demonstrated high levels of BID, as well as considerable anticipated stigma, and perceived loneliness. Specifically, average BID scores exceeded the scores reported during the initial validation study of the BIS with breast cancer patients [48], with 80% of participants reporting scores indicative of clinically significant BID [50]. These findings are consistent with emerging qualitative and quantitative evidence indicating a high prevalence of body image concerns in ILWE [5, 16, 21]. Moreover, the present sample reported experiencing moderate to high endometriosis severity and a range of symptoms, including pelvic pain, abdominal bloating/cramping, fatigue, and nausea/vomiting which likely contributed to the extensive BID reported [5, 21]. Furthermore, consistent with prior qualitative accounts of anticipated appearance-related negative evaluations from others [5], the present study documented relatively high average anticipated stigma scores. These scores were similar to the scale’s original chronic illness sample [52], with each specific source of anticipated stigma (family and friends, work colleagues and healthcare workers) being associated with greater BID. The high perceived severity and anticipated stigma scores may be, in part, due to the study recruiting via a social media support site, as prior research has identified that individuals signing up for such online communities may experience substantially longer diagnostic delays and be the most adversely impacted by their chronic condition [56]. Moreover, many ILWE restrict themselves to only disclosing about their symptoms to others with endometriosis to avoid feeling misunderstood or trivialised, and as such, obtaining a diagnosis may be critical for social connectedness [9, 18, 57].

In terms of loneliness, compared to a large (*N* = 7885) general population sample [58], participants reported similar average social loneliness but greater emotional loneliness, suggesting a greater perceived absence of, or lack of intimacy in, close relationships [59]. This may reflect perceived difficulties arising from the heavy endometriosis symptom burden adversely impacting on the intimate and sexual aspects of close relationships [5, 16]. Australian research has reported that ILWE have difficulties re-engaging socially following the extensive COVID-19 lockdowns that offered them the freedom to manage their symptoms without the pressure of judgement from others [60]. Moreover, since participants were recruited via social media, which can serve to increase feelings of loneliness when utilised to avoid in-person interactions [61], it is perhaps not surprising that emotional loneliness was more prevalent in this study.

Contrary to expectations, the association between BID and greater social loneliness was only significant at the bivariate level. In line with objectification theory regarding BID [30], a possible explanation for this finding is that preoccupation with bodily appearance/functionality may hinder deeper relationships (emotional loneliness) but leave sufficient cognitive resources available to maintain overall connections (social loneliness). Similarly, as evidenced in qualitative investigations, ILWE often reduce their social circles to be more manageable [11] and those experiencing BID may retain relationships with friends with whom they feel less self-conscious about their body image. Hence, this may reduce the experience of body image–related preoccupation in the presence of their social circle [30] and make it easier for the ILWE to maintain those social relationships. Moreover, as objectification theory posits that appearance-related distress is founded in anticipated sexualised evaluation [30], the experience of BID may be more important for intimacy within relationships as opposed to friendships in general.

Indeed, the hypothesised significant association of BID with greater emotional loneliness was supported. Since emotional loneliness reflects aspects of romantic relationships, this is consistent with prior qualitative evidence among ILWE identifying distress anticipating sexual intimacy due to fear of negative body image–related evaluation [5]. Findings in the breast cancer context [62] may shed some light on a possible mechanism explaining the link between BID and emotional loneliness, in that anxiety sharing information about the medical condition was seen as a barrier to connecting with potential romantic partners. Likewise, it may be anxiety discussing symptoms of endometriosis with a romantic partner that underlie the perceived high level of emotional loneliness experienced by participants in the current study. This is additionally supported the absence of a significant association between emotional loneliness and romantic relationship status in the current sample, which may reflect conflict or unmet needs within existing romantic relationships [15].

It was further predicted that anticipated stigma would strengthen the relationship between BID and loneliness; however, this was not supported by the results. Evidence in weight-bias literature may be relevant here as the internalisation of negative weight-related stereotypes is associated with BID [63]. This may be reflected in qualitative accounts of embarrassment and shame regarding endometriosis-related abdominal bloating, which can cause the individual to feel as if they look overweight or pregnant, and in turn drives concealment behaviours [5]. In accordance with objectification theory [30], these internalised negative stereotypes held by ILWE may be more salient and therefore more impactful on determining interpersonal interactions than anticipation of endometriosis-related stigma.

In addition to the relatively high prevalence of emotional loneliness evident, the current sample reported poor perceived social support, as had been reported in prior research among ILWE in Australia [10, 16]. Taken together with the presence of emotional loneliness, this finding highlights the feelings of isolation experienced by ILWE [6]. Perceptions of feeling poorly supported also puts these ILWE at increased risk of developing mental health concerns such as psychological distress [64], and depression and anxiety [65], which, in turn, may compromise their endometriosis self-management [35]. As predicted, greater BID was significantly associated with poorer perceived social support. This is consistent with qualitative investigations among ILWE, indicating a perceived expectation to conceal distress for the sake of maintaining social relationships [11, 33]. According to the practice of self-silencing in ILWE, this suppression of body image–related distress may lead to the individual feeling more distant and less supported by others [11].

Moreover, moderation analyses revealed the detrimental effect that anticipated stigma from healthcare workers can have for ILWE, as this was associated with perceptions of being poorly supported, even in those who are not experiencing significant levels of BID. These findings are consistent with a growing body of qualitative evidence documenting the difficulties that ILWE face in finding a doctor that does not dismiss their symptoms [35, 66]. Healthcare workers have expressed frustration with endometriosis patients and labelled them as ‘difficult’ patients when they continued to express concerns about their condition [67]. As demonstrated in neurological [68] and chronic illness [34] populations, occasions of enacted stigma towards ILWE can lead to the internalisation of this stigma, such that the person believes these negative attitudes about themselves. This internalisation is associated with anticipated stigma [34, 52] which motivates the individual concerned to conceal their symptoms from healthcare professionals [68]. Moreover, anticipated stigma has been associated with self-isolation, which, in itself, is associated with reduced social support [69].

The potentially harmful role of anticipated stigma from healthcare workers identified in this study echoes qualitative evidence highlighting the critical importance of a strong and trustful alliance between patient and doctor for ILWE to potentially improve quality of care and patient psychological outcomes [35, 66]. Additionally, these findings emphasise the need for healthcare professionals to be aware of the psychosocial impacts of endometriosis and ensure ILWE are referred to appropriate services when necessary [35]. To this end, a modified version of the breast cancer intervention, *Restoring Body Image after Cancer* (ReBIC) [70], may be a suitable approach for addressing the needs of ILWE who are experiencing BID. This group-based intervention focuses on addressing BID by normalising experiences in a supportive environment, which has been noted as an important source of validation [70] and may thus additionally reduce feelings of loneliness and isolation. The finding that anticipated stigma from family and friends is associated with social and emotional loneliness brings to light the importance for interventions that focus on perceptions of stigma in the context of social functioning for ILWE. One such intervention is the Australian program *Periods, Pain and Endometriosis* (PPEP Talk) [71] that targets all genders of secondary school–aged children on what constitutes ‘abnormal’ menstrual pain, aiming to improve social support for ILWE by destigmatizing the topic of menstruation [71].

In consideration of these findings, some potential limitations should be noted. Despite recruiting participants from regions across Australia, the sample was, nonetheless, predominantly well-educated, non-Indigenous Australians. Participants were recruited through an online endometriosis community, which may have captured the views of ILWE who are experiencing greater symptoms or difficulties [4, 56]. The cross-sectional design of this study further precludes any causal associations being drawn, highlighting the need for future longitudinal investigations of these variables. Additionally, future research examining perceived social support and anticipated stigma could benefit from including the perspectives of family, friends and healthcare workers to more comprehensively understand the dynamics underlying these negative perceptions in ILWE [62].

In conclusion, this study contributed to the rapidly expanding research documenting psychosocial challenges for ILWE [12, 16, 21], particularly in the domains of social functioning, poor body image and anticipated stigma. Our findings offer insight into the potential adverse impacts of feelings of loneliness and a perceived lack of social support in ILWE, which can be further exacerbated in those additionally experiencing body image disturbance. Moreover, our findings highlight the additional risk factor for adverse outcomes regarding healthcare provider interactions due to the anticipation of stigma related to endometriosis. These findings emphasise the need both for longitudinal research designs to identify the causal pathways and for the development of targeted psychological interventions to address these concerns, beyond medical treatment for symptoms.

### Supplementary Information

Below is the link to the electronic supplementary material.

Supplementary file1 (DOCX 25 KB)

## Data Availability

Deidentified data is available from the corresponding author, upon request.

## Declarations

### Welfare of Animals

This article does not contain any studies with animals performed by any of the authors.
