# Supplementary material for: Loneliness and Perceived Social Support in Endometriosis: The Roles of Body Image Disturbance and Anticipated Stigma
Source: Int J Behav Med. 2023 Oct 26;31(3):433–44. doi: 10.1007/s12529-023-10230-w (PMC11106211; doi:10.1007/s12529-023-10230-w)
Supplement: Supplementary file 1 — Supplementary file1 (DOCX 25 KB) [file 12529_2023_10230_MOESM1_ESM.docx]

Supplementary File

Calvi, Sherman, & Pham (2023)

*Bootstrapped Multivariable Regression Analyses (Including all Interaction Terms)*

| Variables | *b* (*SE b*) | BCA 95% CI for *b* | | *z* | *p* (*z*) | χ^2^ | *Radj*^2^ |
| --- | --- | --- | --- | --- | --- | --- | --- |
|  |  | LL | UL |  |  |  |  |
| Model 1 – Lon_Soc |  |  |  |  |  | 23.97** | .09 |
| Total no. symptoms | 0.06 (0.07) | -0.06 | 0.21 | .94 | .35 |  |  |
| BIS | -0.01 (.04) | -0.10 | 0.07 | -0.33 | .74 |  |  |
| Stigma_FF | 0.32 (0.36) | -0.42 | 1.02 | 0.87 | .39 |  |  |
| Stigma_Work | -0.15 (0.32) | -0.82 | 0.48 | -0.46 | .65 |  |  |
| Stigma_Health | -0.04 (0.26) | -0.55 | 0.46 | -0.17 | .87 |  |  |
| BIS*Stigma_FF | 0.003(.02) | -0.04 | 0.04 | 0.15 | .88 |  |  |
| BIS*Stigma_Work | 0.02(0.02) | -0.02 | 0.05 | 0.96 | .34 |  |  |
| BIS*Stigma_Health | -0.005(0.01) | -0.03 | 0.02 | -0.32 | .75 |  |  |
| Model 2 – Lon_Emot |  |  |  |  |  | 93.21** | .20 |
| Education |  |  |  |  |  |  |  |
| Vocational/Other tertiary education | -0.06 (0.47) | 0.02 | 0.47 | -0.14 | .89 |  |  |
| Undergraduate | -0.46 (0.44) | 0.004 | 0.44 | -1.04 | .30 |  |  |
| Postgraduate | -0.06 (0.49) | 0.01 | 0.49 | -0.13 | .90 |  |  |
| Total no. symptoms | 0.04 (0.09) | -0.12 | 0.22 | 0.46 | .64 |  |  |
| PANAS_Na | 0.02 (0.02) | -0.001 | 0.02 | 1.11 | .27 |  |  |
| BIS | 0.09 (0.06) | -0.02 | 0.20 | 1.67 | .09 |  |  |
| Stigma_FF | 0.19 (0.46) | -0.69 | 1.09 | 0.41 | .68 |  |  |
| Stigma_Work | 0.35 (0.42) | -0.49 | 1.12 | 0.84 | .40 |  |  |
| Stigma_Health | 0.03 (0.37) | -0.71 | 0.75 | 0.08 | .94 |  |  |
| BIS*Stigma_FF | 0.02(0.02) | -0.03 | 0.06 | 0.78 | .43 |  |  |
| BIS*Stigma_Work | -0.01(0.02) | -0.04 | 0.03 | -0.28 | .78 |  |  |
| BIS*Stigma_Health | -0.01(0.02) | -0.05 | 0.02 | -0.80 | .42 |  |  |
| Model 3 –Soc_Support |  |  |  |  |  | 148.86** | .42 |
| Outside metro. area | 3.58(2.71) | -1.75 | 8.95 | 1.32 | .12 |  |  |
| Education |  |  |  |  |  |  |  |
| Vocational/Other tertiary education | 1.11(4.43) | -7.45 | 10.02 | 0.25 | .80 |  |  |
| Undergraduate | -1.96(3.77) | -9.47 | 5.27 | -0.52 | .60 |  |  |
| Postgraduate | -0.20(3.59) | -7.07 | 6.86 | -0.06 | .96 |  |  |
| Employment |  |  |  |  |  |  |  |
| Part time/casual | -1.33(2.93) | -6.05 | 5.46 | -0.05 | .96 |  |  |
| Not working | -2.04(4.08) | -10.73 | 5.30 | -0.50 | .62 |  |  |
| Student/home duties | -4.77(4.40) | -13.44 | 3.87 | -1.09 | .28 |  |  |
| No. of symptoms ^a^ | 0.11(0.80) | -1.36 | 1.78 | 0.14 | .89 |  |  |
| Endometriosis severity^a^ | 0.52(2.56) | -4.28 | 5.45 | 0.20 | .84 |  |  |
| Pain medication ^a^ | 5.24(3.89) | -2.06 | 13.04 | 1.35 | .18 |  |  |
| PANAS_Na ^a^ | 0.33(0.16) | 0.03 | 0.65 | 2.08* | .04 |  |  |
| BIS ^a^ | 1.00(0.22) | 0.57 | 1.43 | 2.57* | .01 |  |  |
| Stigma_FF ^a^ | 0.41(1.77) | -3.04 | 3.77 | -0.27 | .79 |  |  |
| Stigma_Work ^a^ | 2.30(1.48) | -0.68 | 5.03 | 0.32 | .75 |  |  |
| Stigma_Health ^a^ | 4.22(1.30) | 1.74 | 6.84 | 2.64** | .008 |  |  |
| BIS*Stigma_FF ^b^ | 0.13(0.31) | -0.50 | 0.71 | 0.41 | .68 |  |  |
| BIS*Stigma_Work ^b^ | 0.06(0.22) | -0.37 | 0.51 | 0.25 | .80 |  |  |
| BIS* Stigma_Health ^b^ | -0.39(0.20) | -0.77 | 0.005 | -1.91 | .056 |  |  |

*Note.* *SE b*=bootstrapped standard error for *b*; BCA 95% CI= bias-corrected and accelerated 95% confidence interval; LL=lower limit; UL=upper limit; Outside metro. area=living in regional/rural/remote area;

BIS* Stigma_Health =interaction term between BIS and Stigma_Health.

^a^ Numeric variables were mean-centred to aid interpretation of main effects.

^b^ Interaction term constructed using mean-centred variables.

**p* < .05, ***p* < .01
